# Identification and characterization of novel TRPM1 autoantibodies from serum of patients with melanoma-associated retinopathy

**DOI:** 10.1371/journal.pone.0231750

**Published:** 2020-04-23

**Authors:** Juliette Varin, Margaret M. Reynolds, Nassima Bouzidi, Sarah Tick, Juliette Wohlschlegel, Ondine Becquart, Christelle Michiels, Olivier Dereure, Robert M. Duvoisin, Catherine W. Morgans, José-Alain Sahel, Quentin Samaran, Bernard Guillot, José S. Pulido, Isabelle Audo, Christina Zeitz

**Affiliations:** 1 Sorbonne Université, INSERM, CNRS, Institut de la Vision, Paris, France; 2 Department of Ophthalmology, Washington University, Saint Louis, MO, United States of America; 3 CHNO des Quinze-Vingts, DHU Sight Restore, INSERM-DGOS CIC 1423, Paris, France; 4 Department of Dermatology and INSERM U1058 “Pathogenesis and control of chronic infections”, University of Montpellier, Montpellier, France; 5 Department of Chemical Physiology & Biochemistry, Oregon Health & Science University, Portland, OR, United States of America; 6 Fondation Ophtalmologique Adolphe de Rothschild, Paris, France; 7 Académie des Sciences, Institut de France, Paris, France; 8 Department of Ophthalmology, The University of Pittsburgh School of Medicine, Pittsburgh, PA, United States of America; 9 Department of Ophthalmology, Mayo Clinic, Rochester, MN, United States of America; 10 Department of Molecular Medicine, Mayo Clinic, Rochester, MN, United States of America; 11 Institute of Ophthalmology, University College of London, London, United Kingdom; Justus Liebig Universitat Giessen, GERMANY

## Abstract

Melanoma-associated retinopathy (MAR) is a rare paraneoplastic retinal disorder usually occurring in the context of metastatic melanoma. Patients present with night blindness, photopsias and a constriction of the visual field. MAR is an auto-immune disorder characterized by the production of autoantibodies targeting retinal proteins, especially autoantibodies reacting to the cation channel TRPM1 produced in melanocytes and ON-bipolar cells. TRPM1 has at least three different isoforms which vary in the N-terminal region of the protein. In this study, we report the case of three new MAR patients presenting different anti-TRPM1 autoantibodies reacting to the three isoforms of TRPM1 with variable binding affinity. Two sera recognized all isoforms of TRPM1, while one recognized only the two longest isoforms upon immunolocalization studies on overexpressing cells. Similarly, the former two sera reacted with all TRPM1 isoforms on western blot, but an immunoprecipitation enrichment step was necessary to detect all isoforms with the latter serum. In contrast, all sera labelled ON-bipolar cells on *Tprm1*^*+/+*^ but not on *Trpm1*^*-/-*^ mouse retina as shown by co-immunolocalization. This confirms that the MAR sera specifically detect TRPM1. Most likely, the anti-TRPM1 autoantibodies of different patients vary in affinity and concentration. In addition, the binding of autoantibodies to TRPM1 may be conformation-dependent, with epitopes being inaccessible in some constructs (truncated polypeptides versus full-length TRPM1) or applications (western blotting versus immunohistochemistry). Therefore, we propose that a combination of different methods should be used to test for the presence of anti-TRPM1 autoantibodies in the sera of MAR patients.

## Introduction

Paraneoplastic retinopathies are rare retinal disorders usually associated with the presence of autoantibodies against retinal proteins following the development of a primary tumor or a metastasis [1–5]. Two major types of paraneoplastic retinopathies with an initial normal fundus have been reported: (1) cancer-associated retinopathy (CAR), which leads to a rapid and severe visual dysfunction with primary photoreceptor alterations and is most commonly associated with small-cell carcinomas of the lung and less frequently associated with breast, endometrial and other cancers [6,7]; (2) melanoma-associated retinopathy (MAR), traditionally associated with metastatic melanoma [2] but now well recognized in association with other cancers such as carcinomas [8–11]. Patients presenting with MAR usually experience recent night blindness, photopsias (various perception of flickering lights), decreased vision and alterations of the visual field. The fundus examination in patients with MAR is usually normal but may show some degrees of vitritis and vasculitis [12–14]. Cases of disc pallor, vascular attenuation and pigment mottling with time [15] or small choroidal scars [16] have also been reported. The full-field electroretinogram (ff-ERG) is critical for the proper diagnosis of MAR and typically shows ON-bipolar cell dysfunction resembling the ERG abnormalities seen in a sub-group of congenital stationary night blindness (CSNB), the complete form of Schubert-Bornschein, cCSNB [2,17–19]. In this condition, while applying the International Society for Clinical Electrophysiology of Vision (ISCEV) recommended protocol [20], in MAR patients and in cCSNB patients, ff-ERG abnormalities are as follows: under dark adapted (DA, scotopic) conditions, there is no detectable response to a dim (0.01 cd.s.m^-2^) flash. The responses to a bright flash (3.0 and 10.0 cd.s.m^-2^) have an electronegative waveform with a normal negative a-wave, reflecting the normal hyperpolarization of photoreceptors, and severely reduced b-wave in keeping with ON-bipolar cell dysfunction. Light adapted (LA, photopic) responses are also abnormal due to cone-ON-bipolar alterations: a square-shaped a-wave, a sharply arising b-wave and a reduced b/a ration are recorded in response to a single 3.0 cd.s.m^-2^ flash while the 30 Hz response is delayed. Aside mutations in other genes, mutations in *TRPM1* lead to cCSNB [21–30]. The transient receptor potential cation channel subfamily M member 1 (TRPM1) is thought to mediate the depolarization of ON-bipolar cells in response to light, underlying the ERG b-wave [19,31,32]. TRPM1 is not only localized in retinal ON-bipolar cells but also in melanocytes where it plays a role in pigmentation and melanocyte proliferation [33,34]. However, no skin phenotype has been documented in *TRPM1*-related cCSNB [21–23]. Recently, autoantibodies against TRPM1 were identified in patients with MAR, as well as in a few patients with other neoplasms [10,11,35–46]. Despite the fact that anti-TRPM1 autoantibodies have been mostly reported in cases of metastatic melanoma, other groups have also reported patients with carcinoma. Interestingly those also revealed a MAR-like ON-bipolar cell dysfunction on ERG and anti-TRPM1 autoantibodies documented by western blot and immunofluorescence on monkey retina tissue [10,44]. Consequently, as patients with carcinoma can present an ON-bipolar cell dysfunction, a better classification of these two types of paraneoplastic syndromes would be paraneoplastic with photoreceptor defect (usually addressed as CAR) and paraneoplastic with ON-bipolar defect (usually addressed as MAR) [47]. Intravitreal injection of sera from patients with MAR into monkey and mouse eyes resulted in a cCSNB-like ERG phenotype [37,38,48]. TRPM1 localization in ON-bipolar cells, and its implication in cCSNB when mutated, supports the conclusion that paraneoplastic retinopathies showing ON-bipolar cell dysfunctions are caused by autoantibodies binding TRPM1 and blocking its channel function. Whether these anti-TRPM1 antibodies cause other phenotypes such as thinning of the choroid [41] or retinal degeneration is a matter of debate [46,49]. In addition, the ocular phenotype is variable depending on the persistence or not of anti-TRPM1 autoantibodies [44,45]. Indeed, Ueno and colleagues reported clearance of auto-antibodies following treatment in a patient with paraneoplastic retinopathy and ON-bipolar cell dysfunction whose ERG responses did not recover while other patients with MAR or CAR regained normal ERG responses after clearance of anti-TRPM1 antibodies [45] or following rituximab treatment [44], respectively. An interesting hypothesis to explain the occurrence of paraneoplastic retinopathy has been proposed in a case with a primary photoreceptor paraneoplastic retinopathy (referenced there as a case of CAR). Indeed, anti-recoverin antibodies have been identified as reacting to the ectopic expression of recoverin [50]. *TP53*, encoding the tumor suppressor protein P53, is located in the vicinity of recoverin. A mutation in *TP53* would give an aggressive advantage to the tumor, leading to the ectopic expression of recoverin and subsequently the development of autoantibodies cross-reacting with the retinal protein [50]. Similarly, a sequence in intron 8 of *TRPM1* encodes the tumor suppressor miR-211 which is downregulated in aggressive tumor. An alternative splicing would decrease miR-211 expression and lead to aberrant immunogenic TRPM1 promoting the development of autoantibodies [43,51–55]. While auto-antibodies against TRPM1 have been implicated in paraneoplastic ON-bipolar cell dysfunction, further studies have analyzed their specific target. Epitope mapping studies suggest that the autoantibodies target the N-terminal, intracellular domain of TRPM1 [37,43]. Besides, Oancea et al. reported three different *TRPM1* isoforms resulting from alternative splicing (i.e. 70+*TRPM1*, 92+*TRPM1* and 109+*TRPM1*), which differ in the length of their N-terminus, while the transmembrane domains and the C-terminus are conserved [33]. They demonstrated that the three different transcripts of TRPM1 were expressed in the retina using amplification by PCR on retinal cDNA. When expressed in human embryonic kidney (HEK) cells, isoforms 92+*TRPM1* and 109+*TRPM1* are expressed as full-length cation channels as suggested by western blot. However, the 92+*TRPM1* isoform was poorly expressed in the retina while the 70+*TRPM1* was well represented in the retina (no data for the 109+*TRPM1* isoform). Moreover, our group identified a mutation in the exon coding for the 92+*TRPM1* isoform [21]. Duvoisin and colleagues mapped the epitope recognized by MAR sera and defined its coding region from the exons 9 to 10 of *TRPM1* which is common to these three isoforms. To our knowledge, it is not known whether the anti-TRPM1 autoantibodies in MAR patients react differentially towards these distinct N-terminal isoforms. Therefore, in accordance with the literature, the purpose of this study was to detect and further investigate the immunoreactivity of anti-TRPM1 autoantibodies towards the three different isoforms of TRPM1, in three patients with a clinical diagnosis of MAR.

## Material and methods

### Clinical investigation and patient sera

This study was approved by the Comité de Protection des Personnes Ile-de France V and by the institutional review board of the Mayo Clinic, Rochester, MN, USA. All procedures adhered to the Tenets of Helsinki. Three patients with a clinical diagnosis of MAR, two from the Mayo Clinic in Rochester, Minnesota (referred as MAR1 and MAR2) and one from the Centre Hospitalier National d’Ophtalmologie of the Quinze-Vingts hospital, in Paris, France (referred as MAR3) underwent extensive clinical examination to confirm the diagnosis. Testing included: assessment of best corrected visual acuity (BCVA) using the Early Treatment Diabetic Retinopathy Study (EDTRS) chart, kinetic and static perimetry. Full-field electroretinogram (ff-ERG) were performed with DTL recording electrodes and incorporated the ISCEV standard (Veris II for full-field ERG and multifocal ERG for both MAR1 and MAR2 patients while Espion^2^ Diagnosys® was used for ff- ERG and mfERG for the MAR3 patient). Clinical assessment was completed with fundus autofluorescence imaging (FAF) and optical coherence tomography (OCT) (HRAII® and Spectralis® OCT, Heidelberg Engineering, Dossenheim, Germany) [56]. Sera from peripheral blood of the above patients were collected on dry tubes, centrifuged at 2,000 g for 10 minutes and stored at -80°C.

### Animals

Mice were maintained on a 12-hour light-dark cycle. *Trpm1*^*+/+*^ and *Trpm1*^*-/-*^ animals have been previously described by Morgans and colleagues [31]. All experiments were conducted in accordance with National Institutes of Health guidelines and approved by the Institutional Animal Care and Use Committees at Oregon Health & Science University.

### Immunolocalization studies in TRPM1 overexpressing mammalian cells

COS-7 cells, seeded on glass slides coated with 200μg/cm^2^ poly-D-lysine and 1μg/cm^2^ laminin (Sigma-Aldrich, St. Louis, MO, USA), were transfected with plasmids encoding the three different isoforms of human V5-tagged TRPM1 (70*+TRPM1*: 1603 amino acids encoded by 4812 bp, 92+*TRPM1*: 1625 amino acids encoded by 4878 bp and 109+*TRPM1*: 1642 amino acids encoded by 4929 bp; Source Bioscience ImaGenes, Berlin, Germany) using 2 M CaCl_2_ and 2X Hepes Buffered Saline. The medium was changed 24 hours after transfection and immunofluorescent studies were performed 48 hours post-transfection. The V5-tag was present between amino acid residues 807 and 811, 832 and 833, 849 and 850 of each TRPM1 isoform, respectively, a region predicted to be localized extracellularly by *in silico* analysis (UniProtKB-Swiss-Prot). Cells were fixed (AntigenFix, Diapath, Martinengo, Italy) and permeabilized with PBST (PBS, 1% BSA, 0.1% TritonX-100), and proteins were detected with the following primary antibodies: mouse anti-V5 (1:250, Invitrogen, ThermoFisher Scientific, Waltham, MA, USA), rabbit anti-human TRPM1 (1:250, HPA014785, Sigma-Aldrich) and sera from patients (1:250). Primary antibodies were incubated for 1 hour at room temperature and after three PBS washes, slides were incubated with anti-mouse (Alexa Fluor 488), anti-rabbit (Alexa Fluor 488) or anti-human (Alexa Fluor 594) secondary antibodies (1:1000, Jackson ImmunoResearch, West Grove, PA, USA) for 30 minutes at room temperature. Cells were fixed a second time and finally washed with PBS. The slides were then cover-slipped (Vectashield, Vector Laboratories, Burlingame, USA) and air-dried overnight. Fluorescent images of transfected cells were acquired with a confocal microscope (FV1000, Olympus, Hamburg, Germany). Images brightness and contrast were adjusted for publication using the ImageJ Software (Bethesda, MD, USA).

### Preparation of retinal sections

Four *Trpm1*^*+/+*^ and four *Trpm1*^*-/-*^ animals [31] were sacrificed by CO_2_ inhalation followed by cervical dislocation. Eyes were removed and dissected to keep the posterior part of the eyes which were then fixed in ice-cold 4% paraformaldehyde for 20 minutes. Subsequently, eye-cups were washed in ice-cold PBS and cryoprotected by increasing concentrations of sucrose in 0.12 M phosphate buffer (10% and 20% sucrose incubation for 1 hour each at 4°C and 30% sucrose under agitation overnight at 4°C). Subsequently, eye-cups were embedded in 7.5% gelatine—10% sucrose and the blocks frozen at -40°C in isopentane and kept at -80°C until sectioning. Fourteen-μm sections were prepared using a cryostat (MICROM HM 560^™^, ThermoFisher Scientific) and mounted on glass slides (Superfrost® Plus, ThermoFisher Scientific).

### *Ex vivo* immunolocalization studies

Mouse retinal sections were blocked for 1 hour at room temperature in blocking solution (PBS, 10% Donkey Serum (v/v), 0.3% Triton X-100). Primary antibodies and the dilutions used were: human MAR sera (MAR1 1:500, MAR2 1:250 and MAR3 1:250), sheep anti-TRPM1 (1:500; Cao *et al* [57]), and mouse anti-PKCα (1:1,000; P5704 Sigma-Aldrich, Darmstadt, Germany). The sections were incubated with primary antibodies diluted in blocking solution overnight at room temperature. After three washes with PBS, the sections were incubated with anti-human, anti-sheep and anti-mouse secondary antibodies coupled to Alexa Fluor 488, Alexa Fluor 594, or Cy3 (Jackson ImmunoResearch) along with 4’,6-diamidino-2-phenylindole (DAPI), all used at 1:1000, for 1.5 hours at room temperature. Subsequently, the sections were cover-slipped with mounting medium (Mowiol, Merck Millipore, Billerica, MA, USA). Fluorescent images of retinal sections were acquired with a confocal microscope (FV1000, Olympus). Images for figures were adjusted for publication using Image J.

### Western blot analysis

COS-7 cells were transfected with 70+*TRPM1*, 92+*TRPM1*, 109+*TRPM1* plasmids or not transfected. After 48 hours, cells were dissociated by sonication in lysis buffer (Tris 50mM pH7.5, NaCl 150 mM, Triton X-100 1%) along with a combination of anti-protease and anti-phosphatase (Phosphatase Inhibitor Cocktail, Sigma-Aldrich) on ice for 30 minutes. Lysates were cleared by centrifugation at 13,800 x g for 10 minutes at 4°C. Protein extracts from transfected and untransfected cells were separated by SDS-Page on a 4–15% pre-cast gel (Mini-PROTEAN^®^ TGX^™^ Precast Protein Gels, Bio-Rad, Hercules, CA, USA) and subsequently transferred on nitrocellulose membranes (Trans-Blot^®^ Turbo^™^ Midi Nitrocellulose Transfer Packs, Bio-Rad). Membranes were blocked in milk for an hour and then incubated overnight at 4°C with the patients’ sera (1:5,000–1:50,000) or serum from a non-MAR patient (1:50,000) and anti-V5 antibody (1:5,000, Invitrogen, ThermoFisher Scientific). The membranes were then incubated with horseradish peroxidase (HRP)-conjugated donkey anti-human or anti-mouse IgG (1:10,000, Jackson ImmunoResearch) as secondary antibody for 1 hour at room temperature. Bands were revealed using an HRP substrate for chemiluminescence (Pierce^™^ ECL Western Blotting Substrate, ThermoFischer).

### Immunoprecipitation

Following protein extraction, magnetic beads (Dynabeads Protein G, ThermoFischer Scientific) were incubated with 2 μg anti-V5 antibody (Invitrogen, ThermoFisher Scientific) for 1.5 hours at 4°C. Thereafter, tubes containing the beads covered with anti-V5 antibody were placed on a magnet and the unbound antibody containing solution was removed. The protein extract was incubated with the anti-V5-coated magnetic beads overnight at 4°C. Subsequently, beads were washed to remove the excess of protein and re-suspended in Laemmli buffer (2X SDS Urea) at 95°C for 5 minutes. The protein solution was then used for western blotting.

## Results

### ON-bipolar cell dysfunction in three patients with MAR

**Fig 1:**
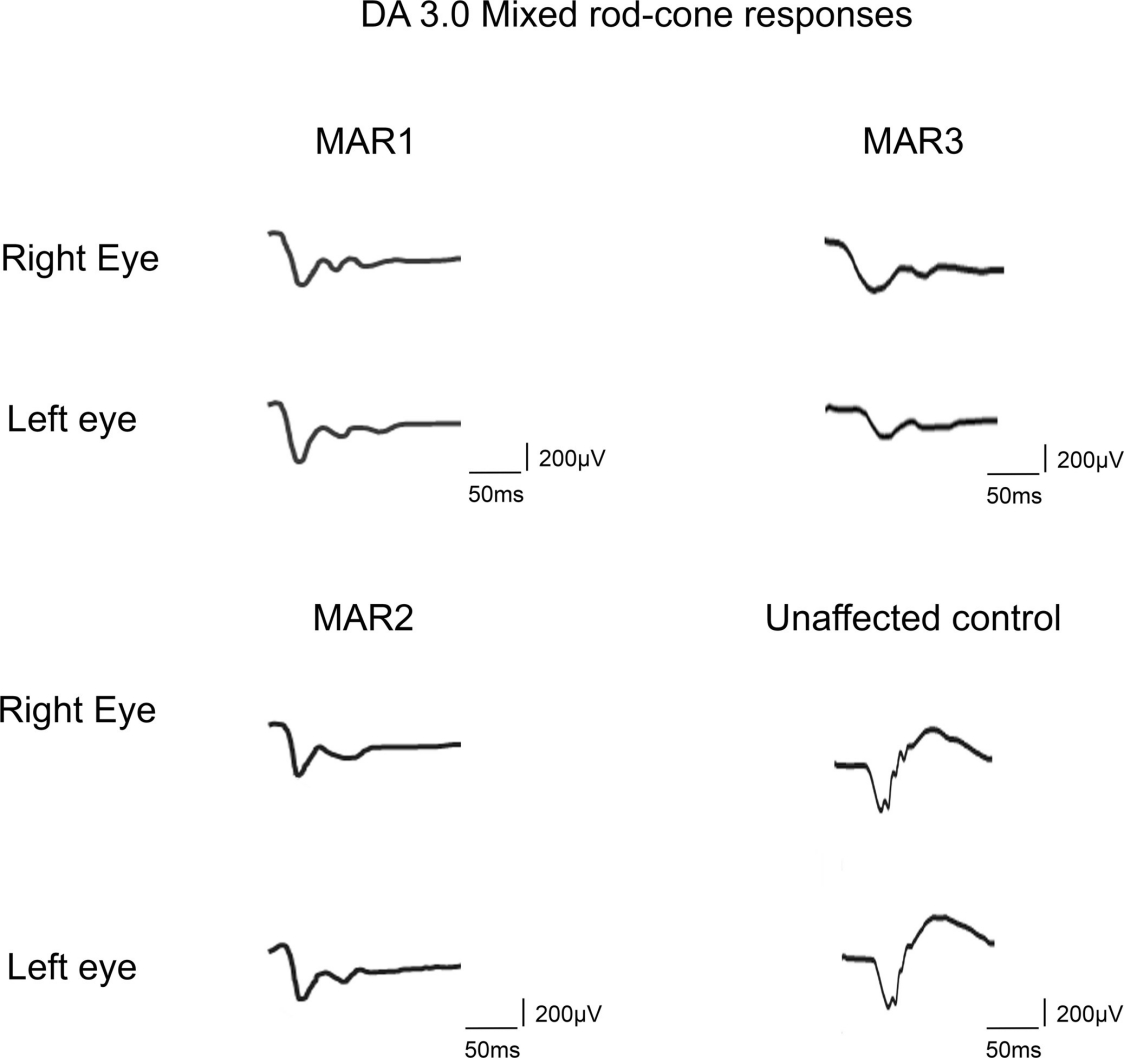
Full-field ERG recordings (DA 3.0).

All patients had biopsy-proven malignant melanoma and ocular findings consistent with classic MAR. Briefly, the first patient (MAR1 patient, Fig 1) was a 63-year-old female with a stage III metastatic malignant melanoma from an unknown primary tumor. She presented with a BCVA of 20/40 in the right eye, which decreased over 1.5 years to 20/200, while the BCVA of 20/40 in the left eye stayed stable. Visual fields showed some mild constriction of the right eye relative to the left eye. The DA 0.01 ERG response was undetectable whereas the DA 3.0 ERG revealed an electronegative response (Fig 1). Photopic responses also showed typical alterations to a single flash with a square-shaped a-wave, a delayed b-wave with however preserved amplitudes as well as a delayed 30 Hz flicker with a squared trough. The photopic ERG was unchanged at the last visit. In addition, this patient revealed also autoantibodies against carbonic anhydrase and enolase.

Ff-ERG responses to a 3.0 cd.s.m^2^ flash under dark-adapted (DA) conditions from an unaffected subject and the three patients with a clinical diagnosis of melanoma-associated retinopathy (MAR). All three patients had a severely reduced b-wave with an electronegative response.

The second patient (MAR2 patient, Fig 1) was a 77-year-old female presenting with a metastatic cutaneous malignant melanoma of the foot with metastases to the right inguinal lymph node and left upper arm. Her BCVA was 20/20 in the right and 20/40 in the left eye. Her visual field appeared constricted bilaterally. In both eyes, the DA 0.01 ERG responses were undetectable with an electronegative response to the DA 3.0 ERG (Fig 1). This patient also displayed a square-shaped a-wave at the photopic ERG responses with a reduced b/a ratio. Multiple autoantibodies were identified in this patient’s serum including anti-GAPDH and anti-enolase antibodies.

The third patient (MAR3 patient, Fig 1) was a 66-year-old female with malignant melanoma of the upper right eyelid that had been excised in 2013. BCVA was 20/160 in the right eye and 20/100 in the left. FAF and spectral domain-OCT were normal. Ff-ERG showed typical ON-bipolar cell dysfunction: there was a severely reduced b-wave to the DA 0.01 ERG responses. The DA 3.0 and DA 10.0 ERG responses showed a normal a-wave but a delayed and severely reduced b-wave leading to an electronegative ERG waveform in accordance with rod-ON bipolar cell dysfunction (Fig 1). Photopic responses were also abnormal with the LA 3.0 ERG showing a square-shaped a-wave with a sharply rising b-wave and a reduced b/a ratio typical of cone-ON bipolar defect.

### Presence of TRPM1 antibodies in all MAR sera as shown by different detection methods

In order to prove the presence of anti-TRPM1 autoantibodies in the sera of these three new cases of MAR, immunolocalization studies on overexpressing cells or mouse retinal cryosections were performed, along with western blot analysis.

### MAR1 and MAR2 sera detect all isoforms of TRPM1, while MAR3 does not react with the short isoform of TRPM1 by immunolocalization studies in overexpressing cells

**Fig 2:**
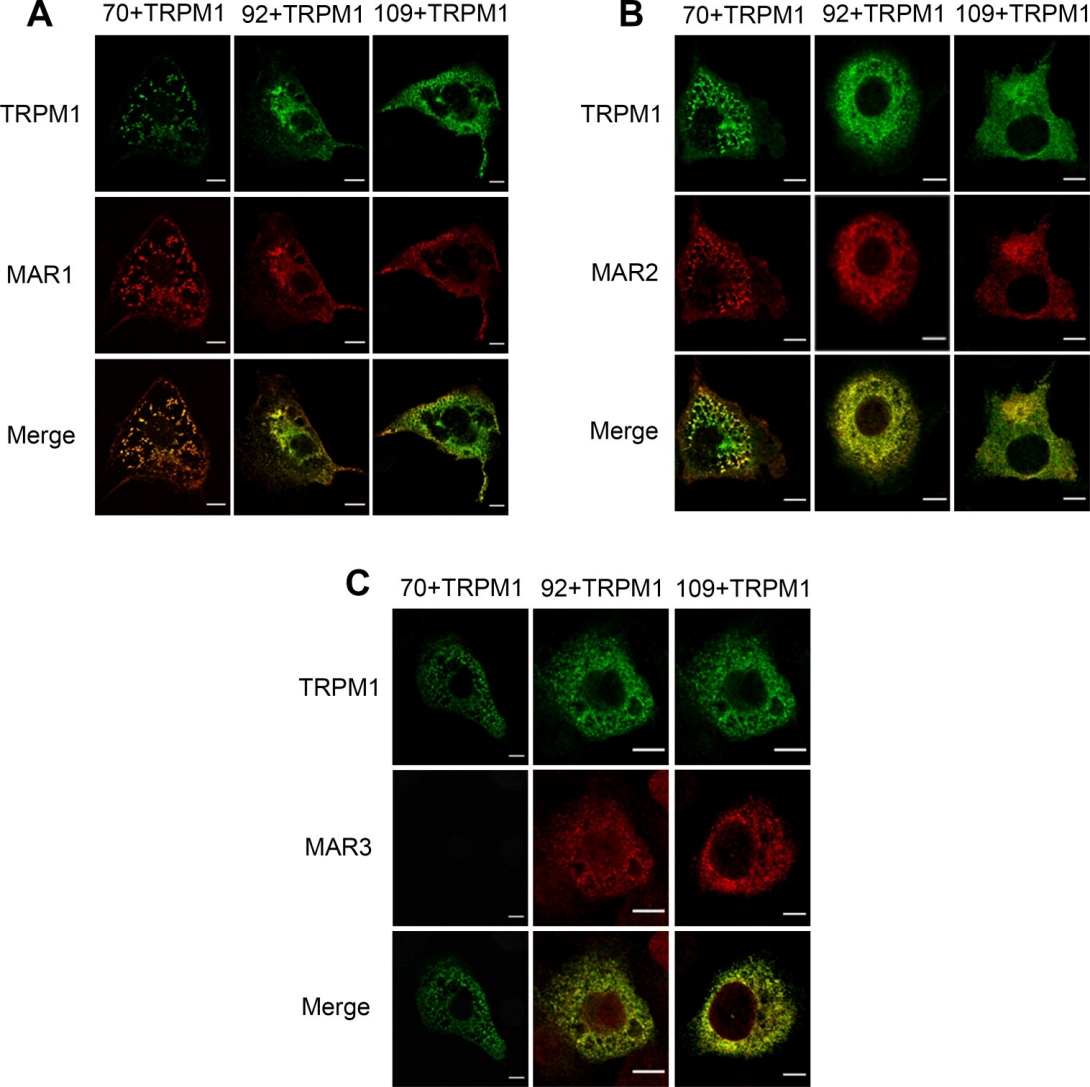
Immunolocalization studies using the MAR sera and anti-TRPM1 antibody in COS-7 cells overexpressing the three isoforms of TRPM1. (A) MAR1 (red) staining colocalized (yellow, merge) with TRPM1 (green) staining in COS-7 overexpressing all three isoforms of TRPM1. (B) MAR2 (red) staining colocalized (yellow, merge) with TRPM1 (green) staining in COS-7 overexpressing all three isoforms of TRPM1. (C) MAR3 (red) staining colocalized (yellow, merge) with TRPM1 (green) staining only in COS-7 overexpressing the 92+*TRPM1* and the 109+*TRPM1* isoform. Scale bars: 10**μ**m.

Because of the previous findings that autoantibodies against TRPM1 were found in the serum of patients with MAR, the sera of the three MAR patients (MAR1-3) reported herein were tested on COS-7 cells overexpressing the different isoforms of human TRPM1 (70+*TRPM1*, 92+*TRPM1* and 109+*TRPM1*). While MAR1 and MAR2 revealed typical TRPM1 staining for all three different isoforms as shown by co-staining with a commercially available antibody against TRPM1 (Fig 2A and 2B), MAR3 labelled the 92+*TRPM1* and the 109+*TRPM1* isoforms, but did not detect the shortest isoform of TRPM1: 70*+TRPM1* (Fig 2C).

### MAR1, MAR2 and MAR3 sera detect all isoforms of TRPM1 by western blot analysis

**Fig 3:**
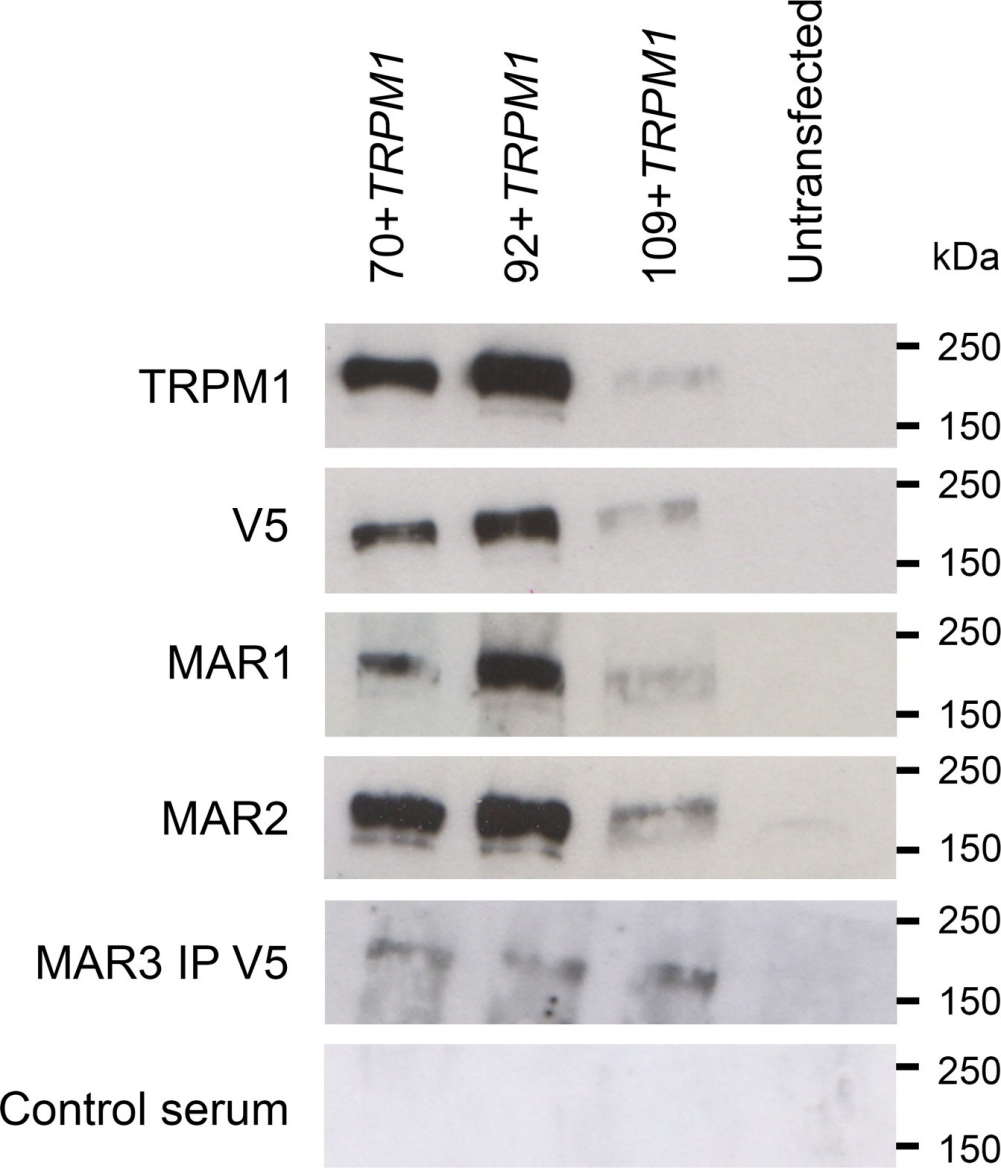
Western blot analysis of TRPM1 isoforms with MAR sera.

MAR sera were tested for their ability to detect TRPM1 following the over-expression of the three different isoforms of V5-tagged human TRPM1 (70+*TRPM1*, 92+*TRPM1* and 109+*TRPM1*) in COS-7 cells by western blot analyses. As expected, MAR1 and MAR2 sera both detected all three isoforms of TRPM1 at the expected size of about 180 kDa (Fig 3). MAR1 and MAR2 displayed comparable reactivity to 109+TRPM1 as the commercial V5 and TRPM1 antibodies, indicating that the fainter bands for this sample are due to lower expression of the longest isoform. The serum of a non-MAR patient did not lead to any staining. Under the same conditions, the MAR3 serum staining was inconclusive, as no bands were detected. Thus, to obtain a specific staining, protein extracts from COS-7 cells over expressing the three TRPM1 isoforms were immunoprecipitated with the anti-V5 antibody. Subsequently, the MAR3 serum detected all three isoforms of TRPM1. Thus, by western blot analyses, the three MAR sera reacted with all three different TRPM1 isoforms.

Immuno blots of COS-7 cells transfected with the three isoforms of TRPM1 using several antibodies: an anti-TRPM1 antibody, an anti-V5 antibody, MAR1 and MAR2 sera, MAR3 sera after immunoprecipitation using an anti-V5 antibody and a control serum. All antibodies recognized the 70+*TRPM1*, 92+*TRPM1* and 109+*TRPM1* isoforms (~180-200kDa). No signal was obtained with protein extracted from untransfected cells.

### MAR1, MAR2 and MAR3 sera stain ON-bipolar cells on mouse retinal cryosections

**Fig 4:**
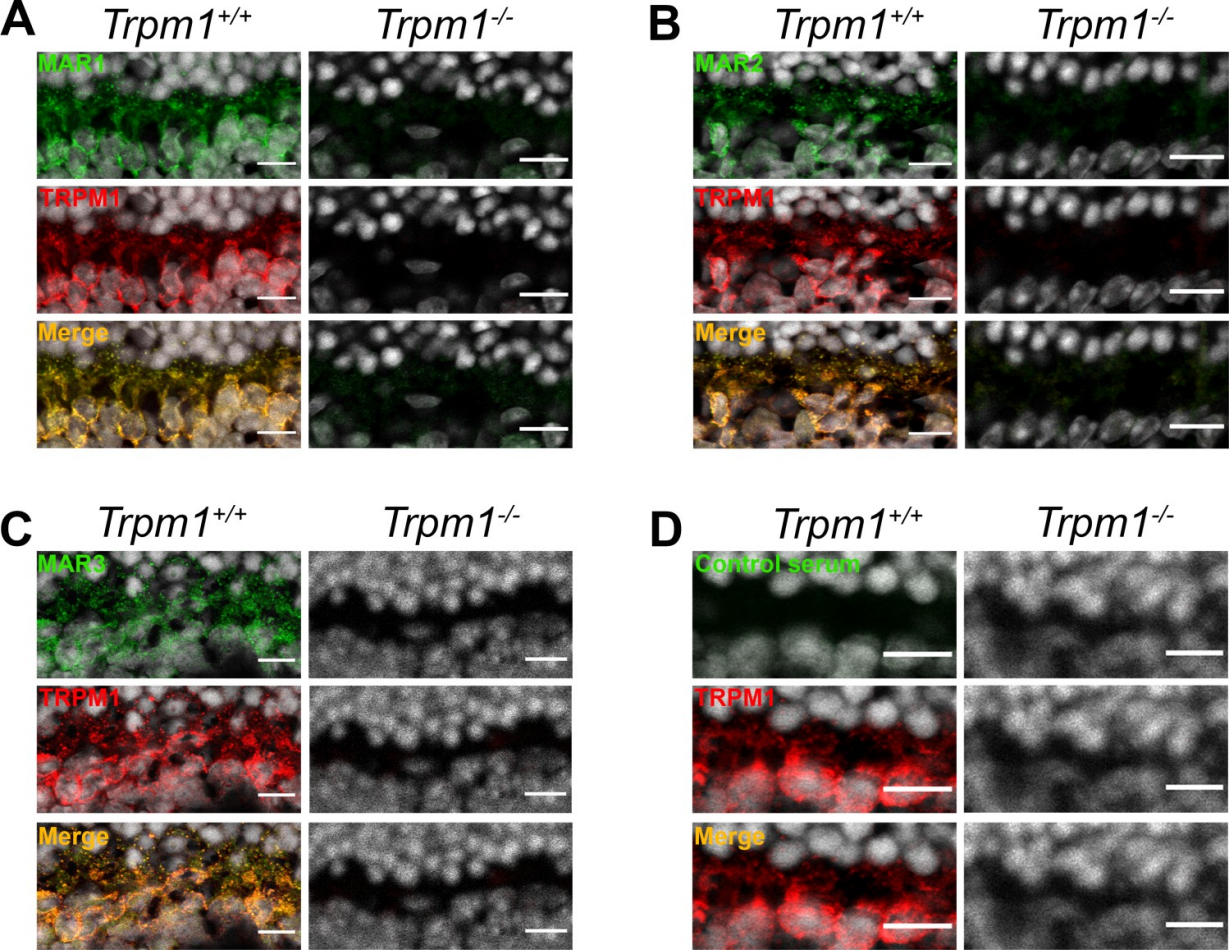
Immunolocalization on *Trpm1*^*+/+*^ and *Trpm1*^*-/-*^ mouse retinal cryosections. (A) TRPM1 (red) staining colocalized (yellow, merge) with MAR1 (green) in *Trpm1*^*+/+*^ and both staining were absent in *Trpm1*^*-/-*^ mouse. (B) TRPM1 (red) staining colocalized (yellow, merge) with MAR2 (green) in *Trpm1*^*+/+*^ and both staining were absent in *Trpm1*^*-/-*^ mouse. (C) TRPM1 (red) staining colocalized (yellow, merge) with MAR3 (green) in *Trpm1*^*+/+*^ and both staining were absent in *Trpm1*^*-/-*^ mouse. (D) No TRPM1 (red) staining was observable in *Trpm1*^*+/+*^ and *Trpm1*^*-/-*^ mouse and the same observation was made with a control serum. Scale bars: 10μm.

To further confirm the results obtained *in vitro*, we tested the three sera on wild-type (*Trpm1*^+/+^) and knock-out *TRPM1* (*Trpm1*^-/-^) mouse retinal cryosections. As expected, the three sera reacted with a protein localized at the dendritic tips and around ON-bipolar cells (Fig 4A, 4B and 4C). This immunolabelling of dendritic tips and around the soma of ON-bipolar cells with all three sera was comparable to the one obtained with an anti-TRPM1 antibody, while such staining was absent using a serum from a subject with no ocular disorder (Fig 4D). This staining was absent in *Trpm1*^-/-^ mice confirming the presence of TRPM1-dependent autoantibodies in all three sera. Immunolabelling was also observed in other retinal layers with these three sera in both wild-type and *Trpm1*^-/-^ mice, most likely due to autoantibodies not directed towards TRPM1 (S1 Appendix).

## Discussion

In this study, we report three novel cases with MAR being in fact females. It has been suggested that MAR is more prevalent in male [8], due to melanoma being more prevalent in male patients, especially after 50 years [58,59]. However, our studied cases were all female patients. We are not aware about the presence of a higher frequency of male or female patients with MAR investigated in the respective hospitals. Interestingly it was previously shown that the average age of patients affected with paraneoplastic retinopathy is 62 [60], which is consistent with our study. Indeed, our patients were between 63 and 77 years old.

The presence of anti-TRPM1 autoantibodies in the sera of these new cases of MAR was validated in mammalian cells overexpressing TRPM1 by immunolocalization, western blot analysis and by immunofluorescence on mouse retinal cryosections. The human TRPM1 exists in three isoforms which differ in their N-terminus [33]. Therefore, we investigated the reactivity of the anti-TRPM1 autoantibodies of our MAR patients towards all TRPM1 isoforms. All three sera contained antibodies reacting to the three isoforms of TRPM1, albeit with different apparent sensitivity. In previous reports, it has been suggested that all anti-TRPM1 autoantibodies in MAR patients recognize the same epitope located at the N-terminal, intracellular region of the protein encoded by exons 9 and 10, which is common to all three isoforms [37,43]. For our MAR patients, anti-TRPM1 autoantibodies recognized an epitope that is common to all three TRPM1 isoforms, encoded some place after exon 2, consistent with the literature, but the sensitivity to detect TRPM1 was different and was method-dependent. This was especially true for the anti-TRPM1 autoantibodies present in MAR3, which failed to label the shortest isoform of TRPM1 in the overexpressing cell system by immunofluorescence, yet detected it by western blot after enrichment, and labelled TRPM1 robustly on mouse retinal sections.

Several hypotheses can be proposed to explain this variability: the epitope recognized by the autoantibodies may vary between patients either because of the tumor type or their immune system specificities or both. In addition, the protein folding may be different depending on the TRPM1 isoforms, not allowing the anti-TRPM1 autoantibodies of the MAR3 patient to bind to the same epitope as in MAR1 and MAR2. Variability in binding might also be explained by distinct antibody titers towards specific isoforms or differences in antibody affinity. As various results were obtained with the same serum depending on the experiment, we suggest to combine multiple tests to assess with certainty the presence of anti-TRPM1 autoantibodies even though in a study by Dalal *et al*., immunofluorescence on tissue sections and cells were sufficient to validate the presence of anti-TRPM1 autoantibodies in a patient with MAR [39]. In our opinion, western blotting should be the first test to be performed to decipher the presence or absence of anti-TRPM1 as the denaturing conditions might promote epitope accessibility for the autoantibodies. Immunoprecipitation prior to western blotting should also be considered as the titer of the autoantibodies in the sera may vary. Finally, if the western blot analysis is inconclusive, other tests, as immunolocalization, should be performed.

In patients affected with MAR, the ERG phenotype is similar to those found in cCSNB, with a severely decreased b-wave, indicating an ON-bipolar cell dysfunction [33,34] as present in the subjects reported in this study. Other laboratories have studied the impact of intravitreal injection of sera from patients with paraneoplastic retinopathy on mouse [37,38] or monkey ERGs [48]. Ueno and colleagues [38] described a reduction of the scotopic b-wave with a relative preserved a-wave, as was also observed by Xiong et al [37]. Similarly, on the monkey ERG, the serum suppressed the photopic b-wave, but the effect on the a-wave was similar to the one obtained with a non-MAR serum [48]. The variable sensitivity in the three sera to detect TRPM1 herein could have also different consequences of the clinical course of the patients ERGs, as some patients recovered after diagnosis and some others deteriorated as previously described [45]. However, a follow-up of the patients studied herein is not available.

Of note, paraneoplastic syndromes can sometimes be more complex due to the occurrence of multiple autoantibodies in the same patient [16]. This may explain additional retinal alterations reported in MAR patients such as choroidal atrophy [41] and MAR patients with other clinical abnormalities [15] which may not be solely explained by anti-TRPM1 antibodies. However, as anti-TRPM1 autoantibodies are the most commonly reported autoantibodies in cases of MAR [35], the search for these other retinal autoantibodies might not have been done. The sera of our three MAR patients were found to specifically label not only the dendritic tip of ON-bipolar cells in wild-type mouse but also other retinal layers in both, wild-type and TRPM1 knock-out mouse retina, suggesting the presence of autoantibodies to multiple retinal targets. The sera of MAR1 and MAR2 patients have been tested for other autoantibodies and were positive for anti-enolase, anti-carbohydrase, and anti-GAPDH antibodies. The presence of anti-α enolase has been associated with normal to severe cone loss [61]. It is however unclear if this autoantibody had a functional impact on the clinical presentation of our patient. Additionally, anti-carbonic anhydrase autoantibodies have been studied by Adamus et al., showing their detrimental effect on a retinal cell line, leading to cell death [62]. To our knowledge, the role of anti-GAPDH autoantibodies remains unclear. Furthermore, the identification and characterization of autoantibodies responsible of the retinal phenotype of patients with paraneoplastic syndromes with either photoreceptors defect or ON-bipolar cell defect can help to establish the proper treatment for these patients. For instance, Roels and colleagues [44] reported the case of a patient with CAR and presenting anti-TRPM1 autoantibodies. Following the treatment of this patient with rituximab in order to decrease the immune response, the serology proved the clearance of the anti-TRPM1 autoantibodies. This led to a normalization of the ERG and an improvement of the symptoms in this patient.

The conclusion of our study is that it might be crucial to use different methods to determine which antigen is recognized by the autoantibodies present in MAR serum. Indeed, a combination of *in vitro* and *ex vivo* methods seems to be the most efficient way to identify autoantibodies in the serum of MAR patients. Moreover, multiple autoantibodies can be implicated in the phenotype of patients with autoimmune retinopathy.

## Supporting information

S1 Appendix Immunolocalization on *Trpm1*^*+/+*^ and *Trpm1*^*-/-*^ mouse retinal cryosections. Sera from MAR patients (green) stain ON-bipolar cells along with a fainter staining in the inner plexiform layer and the ganglion cell layer in both *Trpm1*^*+/+*^ and *Trpm1*^*-/-*^ animals. Scale bars: 10μm.
(TIF)

S1 Raw Images (PDF)
