# Correction: Identification and characterization of novel TRPM1 autoantibodies from serum of patients with melanoma-associated retinopathy

**DOI:** 10.1371/journal.pone.0233424

**Published:** 2020-05-13

**Authors:** The PLOS ONE Staff

The following information is missing from the Funding statement: JV, NB, JW, CZ, IA, JAS: IHU FOReSIGHT (ANR-18-IAHU-0001) supported by French state funds managed by the Agence Nationale de la Recherche within the Investissements d'Avenir program.

The legends for Figs 1 and 3 mistakenly appear in the body of the article. There is an error in the legend for panel B of Fig 4. Please see the complete, correct legends for Figs 1, 3 and 4 here.

**Fig 1:**
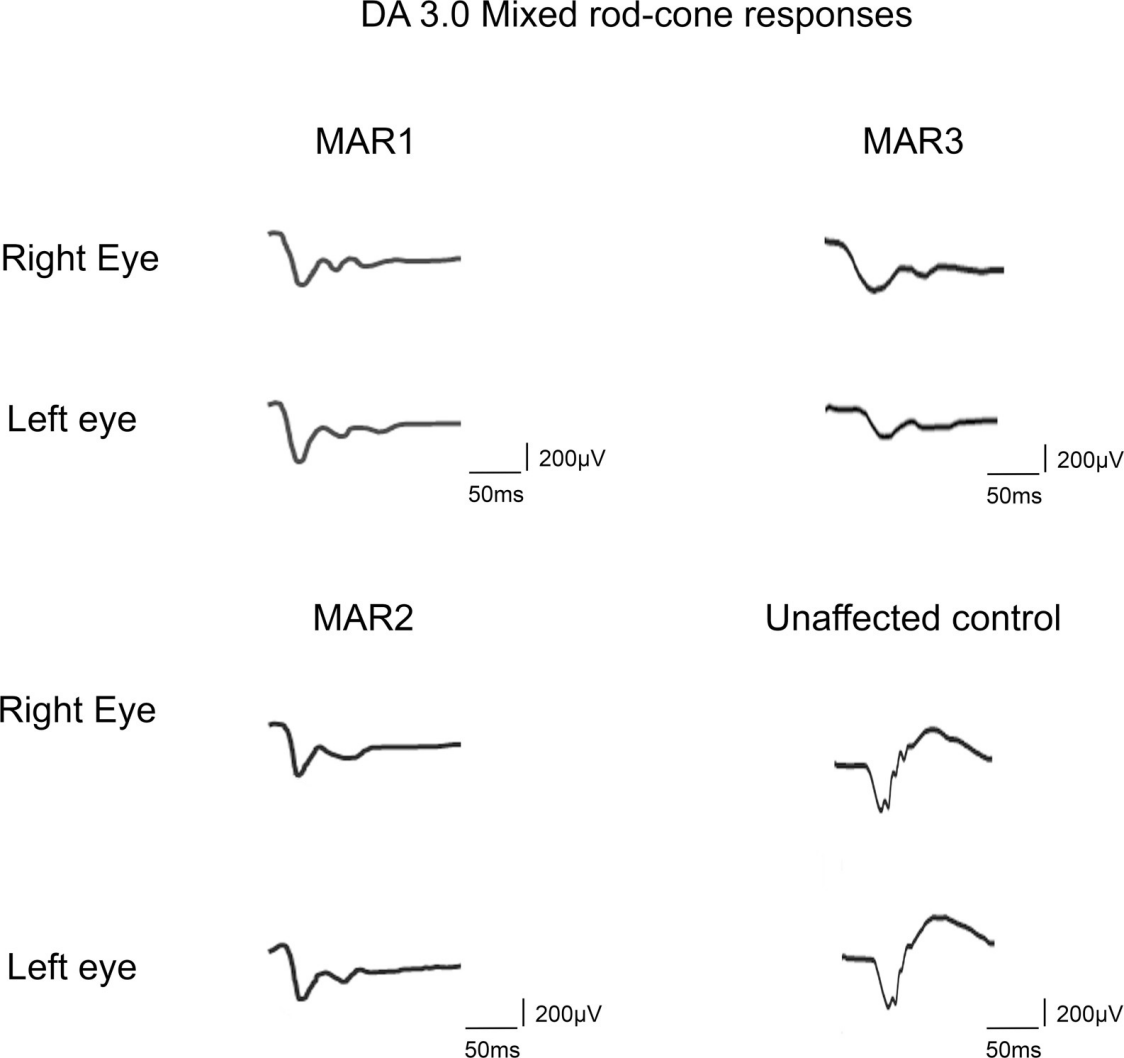
Full-field ERG recordings (DA 3.0). Ff-ERG responses to a 3.0 cd.s.m^2^ flash under dark-adapted (DA) conditions from an unaffected subject and the three patients with a clinical diagnosis of melanoma-associated retinopathy (MAR). All three patients had a severely reduced b-wave with an electronegative response.

**Fig 3:**
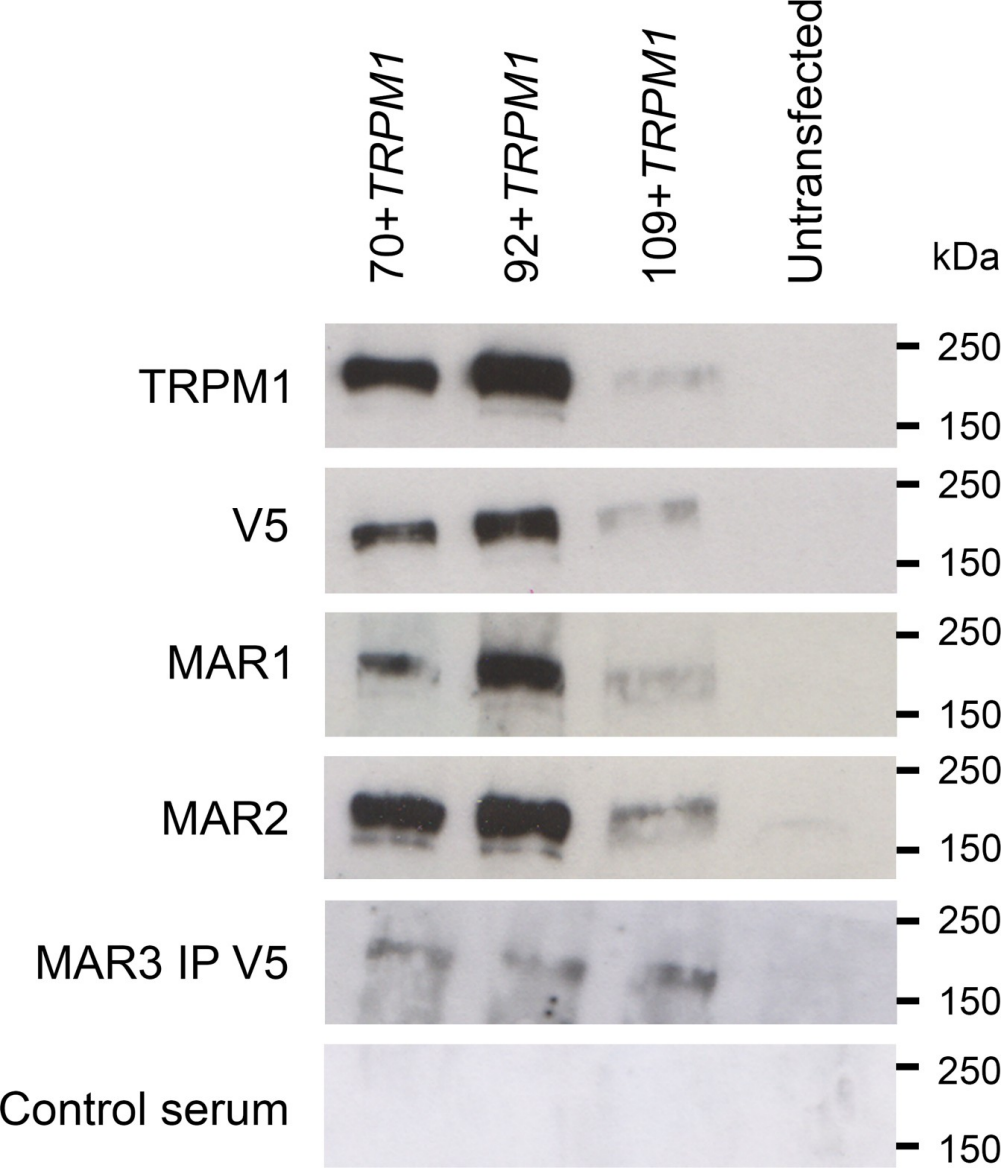
Western blot analysis of TRPM1 isoforms with MAR sera. Immuno blots of COS-7 cells transfected with the three isoforms of TRPM1 using several antibodies: an anti-TRPM1 antibody, an anti-V5 antibody, MAR1 and MAR2 sera, MAR3 sera after immunoprecipitation using an anti-V5 antibody and a control serum. All antibodies recognized the 70+*TRPM1*, 92+*TRPM1* and 109+*TRPM1* isoforms (~180-200kDa). No signal was obtained with protein extracted from untransfected cells.

**Fig 4:**
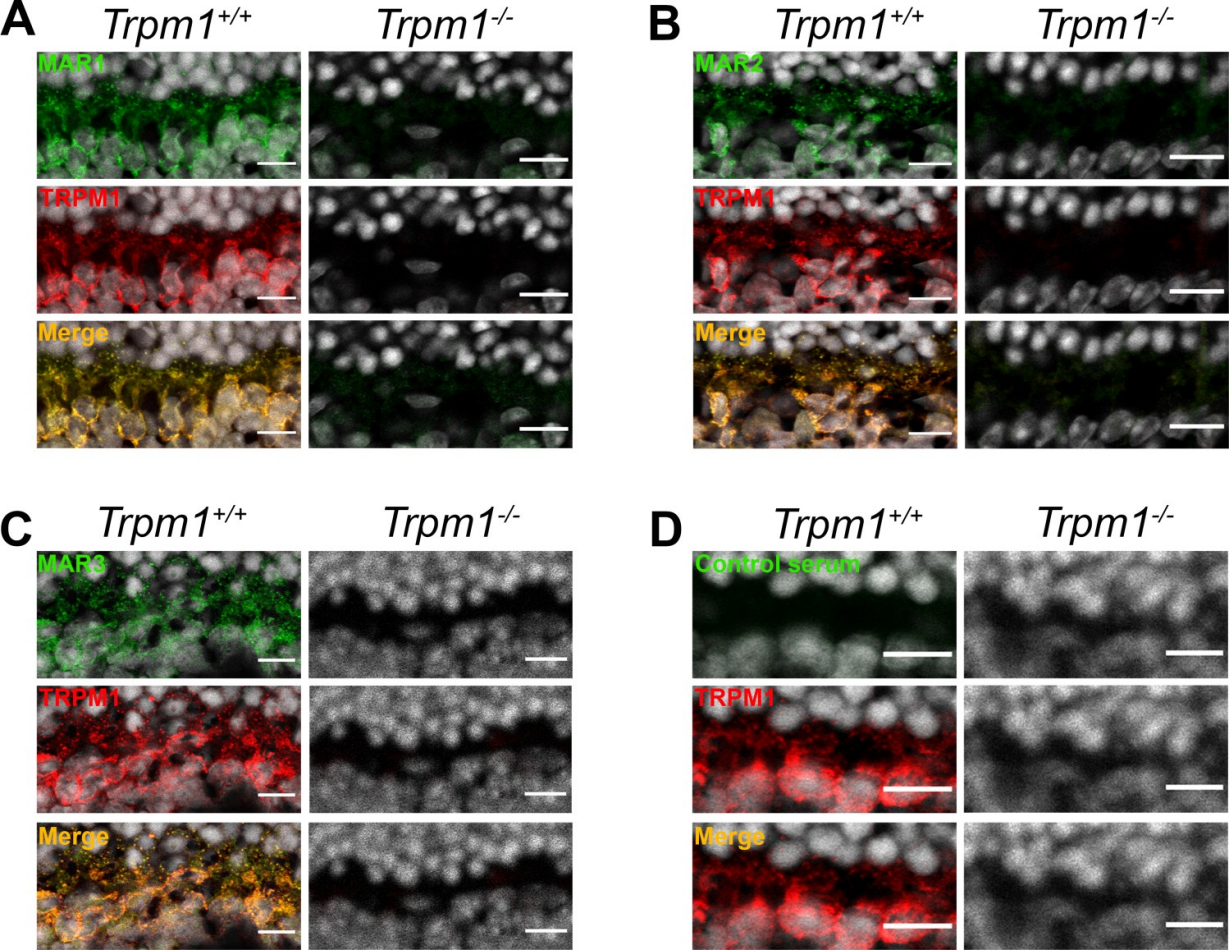
Immunolocalization on *Trpm1*^*+/+*^ and *Trpm1*^*-/-*^ mouse retinal cryosections. (A) TRPM1 (red) staining colocalized (yellow, merge) with MAR1 (green) in *Trpm1*^*+/+*^ and both staining were absent in *Trpm1*^*-/-*^ mouse. (B) TRPM1 (red) staining colocalized (yellow, merge) with MAR2 (green) in *Trpm1*^*+/+*^ and both staining were absent in *Trpm1*^*-/-*^ mouse. (C) TRPM1 (red) staining colocalized (yellow, merge) with MAR3 (green) in *Trpm1*^*+/+*^ and both staining were absent in *Trpm1*^*-/-*^ mouse. (D) TRPM1 (red) staining was present in *Trpm1*^*+/+*^ and absent in *Trpm1*^*-/ -*^mice. Control serum did not reveal any staining. Scale bars: 10μm.

The citation for reference 20 is incomplete. The complete reference is: McCulloch DL, Marmor MF, Brigell MG, Hamilton R, Holder GE, et al. (2015) ISCEV Standard for full-field clinical electroretinography (2015 update). Doc Ophthalmol 130: 1–12. pmid: 25502644

The publisher apologize for the errors.
