# Supplementary figures and images for: Identification and characterization of novel TRPM1 autoantibodies from serum of patients with melanoma-associated retinopathy
Source: PLoS One. 2020 Apr 23;15(4):e0231750. doi: 10.1371/journal.pone.0231750 (PMC7179873; doi:10.1371/journal.pone.0231750)

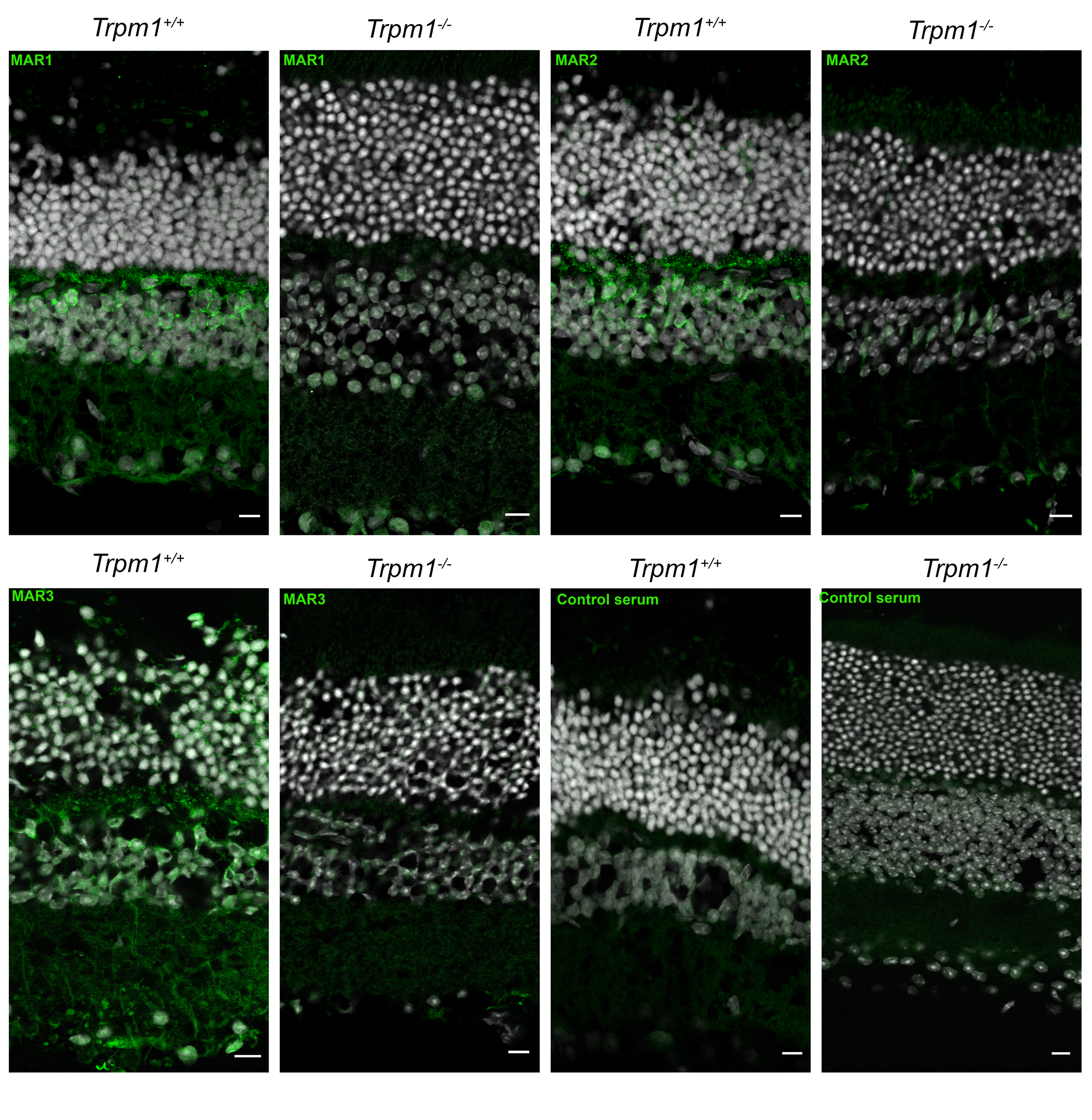

Supplement: S1 Appendix — Sera from MAR patients (green) stain ON-bipolar cells along with a fainter staining in the inner plexiform layer and the ganglion cell layer in both Trpm1+/+ and Trpm1-/- animals. Scale bars: 10μm. (TIF) [file pone.0231750.s001.tif]
