# Supplementary material for: Identification and characterization of novel TRPM1 autoantibodies from serum of patients with melanoma-associated retinopathy
Source: PLoS One. 2020 Apr 23;15(4):e0231750. doi: 10.1371/journal.pone.0231750 (PMC7179873; doi:10.1371/journal.pone.0231750)
Supplement: S1 Raw Images — (PDF) [file pone.0231750.s002.pdf]

Scanned western blot films revealed with ECL (ThermoFischer)

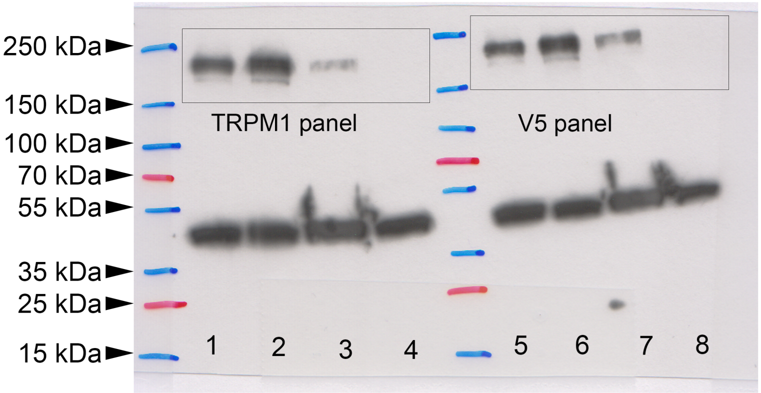

1 : 70+*TRPM1* labelled with anti-TRPM1
2 : 92+*TRPM1* labelled with anti-TRPM1
3 : 109+*TRPM1* labelled with anti-TRPM1
4 : Untransfected COS-7 protein extract labelled with anti-TRPM1
5 : 70+*TRPM1* labelled with anti-V5
6 : 92+*TRPM1* labelled with anti-V5
7 : 109+*TRPM1* labelled with anti-V5
8 : Untransfected COS-7 protein extract labelled with anti-V5

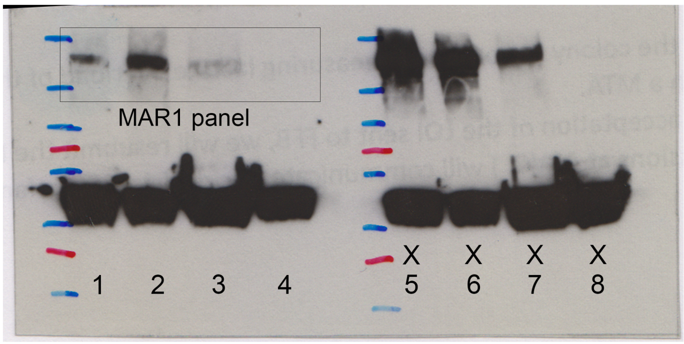

1 : 70+*TRPM1* labelled with MAR1 serum
2 : 92+*TRPM1* labelled with MAR1 serum
3 : 109+*TRPM1* labelled with MAR1 serum
4 : Untransfected COS-7 protein extract labelled with MAR1 serum
5 : 70+*TRPM1* labelled with anti-V5
6 : 92+*TRPM1* labelled with anti-V5
7 : 109+*TRPM1* labelled with anti-V5
8 : Untransfected COS-7 protein extract labelled with anti-V5

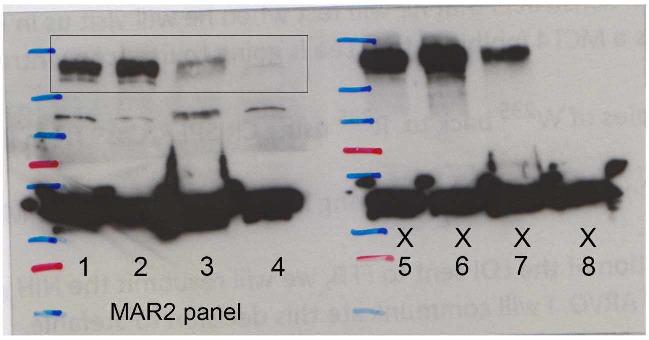

1 : 70+*TRPM1* labelled with MAR2 serum
2 : 92+*TRPM1* labelled with MAR2 serum
3 : 109+*TRPM1* labelled with MAR2 serum
4 : Untransfected COS-7 protein extract labelled with MAR2 serum
5 : 70+*TRPM1* labelled with anti-V5
6 : 92+*TRPM1* labelled with anti-V5
7 : 109+*TRPM1* labelled with anti-V5
8 : Untransfected COS-7 protein extract labelled with anti-V5

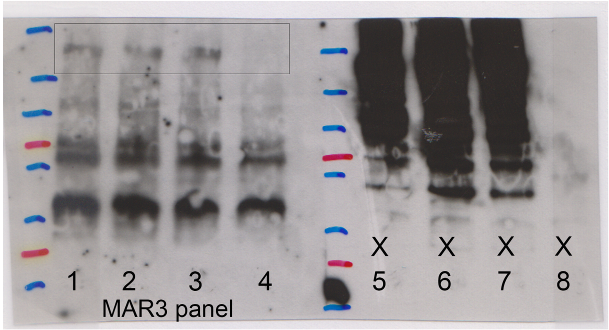

1 : 70+*TRPM1* labelled with MAR3 serum after IP
2 : 92+*TRPM1* labelled with MAR3 serum after IP
3 : 109+*TRPM1* labelled with MAR3 serum after IP
4 : Untransfected COS-7 protein extract labelled with MAR3 serum after IP
5 : 70+*TRPM1* labelled with anti-TRPM1 after IP
6 : 92+*TRPM1* labelled with anti-TRPM1 after IP
7 : 109+*TRPM1* labelled with anti-TRPM1 after IP
8 : Untransfected COS-7 protein extract labelled with anti-TRPM1 after IP

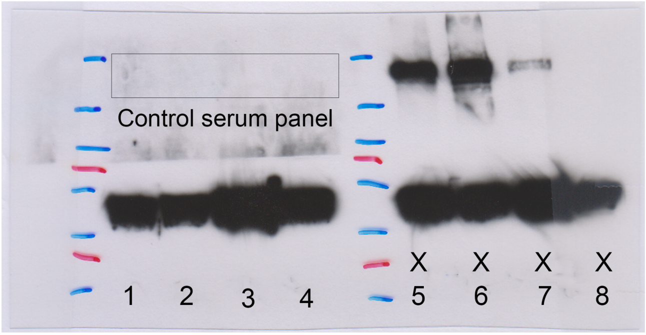

1 : 70+*TRPM1* labelled with control serum
2 : 92+*TRPM1* labelled with control serum
3 : 109+*TRPM1* labelled with control serum
4 : Untransfected COS-7 protein extract labelled with control serum
5 : 70+*TRPM1* labelled with anti-V5
6 : 92+*TRPM1* labelled with anti-V5
7 : 109+*TRPM1* labelled with anti-V5
8 : Untransfected COS-7 protein extract labelled with anti-V5
